# CRISPR/Cas9-mediated efficient genome editing via blastospore-based transformation in entomopathogenic fungus *Beauveria bassiana*

**DOI:** 10.1038/srep45763

**Published:** 2017-04-03

**Authors:** Jingjing Chen, Yiling Lai, Lili Wang, Suzhen Zhai, Gen Zou, Zhihua Zhou, Chunlai Cui, Sibao Wang

**Affiliations:** 1 CAS Key Laboratory of Insect Developmental and Evolutionary Biology, Institute of Plant Physiology and Ecology, Shanghai Institutes for Biological Sciences, Chinese Academy of Sciences, Shanghai 200032, China; 2 University of Chinese Academy of Sciences, Beijing 100049, China; 3 CAS Key Laboratory of Synthetic Biology, Institute of Plant Physiology and Ecology, Shanghai Institutes for Biological Sciences, Chinese Academy of Sciences, Shanghai 200032, China

## Abstract

*Beauveria bassiana* is an environmentally friendly alternative to chemical insecticides against various agricultural insect pests and vectors of human diseases. However, its application has been limited due to slow kill and sensitivity to abiotic stresses. Understanding of the molecular pathogenesis and physiological characteristics would facilitate improvement of the fungal performance. Loss-of-function mutagenesis is the most powerful tool to characterize gene functions, but it is hampered by the low rate of homologous recombination and the limited availability of selectable markers. Here, by combining the use of uridine auxotrophy as recipient and donor DNAs harboring auxotrophic complementation gene *ura5* as a selectable marker with the blastospore-based transformation system, we established a highly efficient, low false-positive background and cost-effective CRISPR/Cas9-mediated gene editing system in *B. bassiana*. This system has been demonstrated as a simple and powerful tool for targeted gene knock-out and/or knock-in in *B. bassiana* in a single gene disruption. We further demonstrated that our system allows simultaneous disruption of multiple genes via homology-directed repair in a single transformation. This technology will allow us to study functionally redundant genes and holds significant potential to greatly accelerate functional genomics studies of *B. bassiana*.

Insect pathogenic fungi are important natural regulators of pest insect populations. Particularly, those belonging to the genera *Beauveria* and *Metarhizium* have shown great promise as biological control agents of insect pests^1^,^2^,^3^. *B. bassiana* has been mass-produced commercially and provides many advantages as an environmentally friendly alternative to chemical insecticides. This fungus is also a facultative saprophyte and can grow as a plant endophyte that confers protection against marauding insects and diseases^4^. It has been widely used to control various agricultural insect pests and vectors of human diseases, including mosquitoes, flies and ticks^5^,^6^,^7^. Moreover, *B. bassiana* has been used as a model system to study fungal development and host-pathogen interactions^8^. However, its field application has been limited due to slow kill and sensitivity to abiotic stresses such as UV radiation, high temperature, drought, which lead to inconsistent results in field application^9^. But genetic engineering has been proved to be a promising strategy to significantly improve fungal virulence, increase stress tolerance, and reduce transmission of mosquito-borne diseases^10^,^11^,^12^. This highlights the need for a better understanding of virulence determinants and physiological characteristics in *B. bassiana*. In this regard, the reverse genetics, involving targeted disruption of candidate genes, remains the most powerful tool to study gene functions and molecular basis of fungal pathogenesis.

The major limitation for gene disruption in *B. bassiana*, and indeed most filamentous fungi, has been the relatively low rate (lower than 5%) of homologous recombination, mainly due to the presence of nonhomologous end joining (NHEJ) DNA repair pathways that often result in the ectopic insertion of the targeting DNA^13^. Currently, the most widespread method for generation of mutants in *B. bassiana* is based on *Agrobacterium*-mediated transformation^14^,^15^,^16^,^17^. However, the *Agrobacterium*-mediated gene targeting method is technically complicated, laborious, together with low rates of homologous recombination and we usually need to screen hundreds to thousands of transformants across multiple transformation attempts to obtain the desired mutant^18^.

Another limitation for mutagenesis in *B. bassiana* is the lack of adequate dominant selectable markers. Most *B. bassiana* strains appear to display intrinsic resistant to most commonly used antibiotic-selective markers. Currently, only two predominant drug resistance markers, the phosphinothricin acetyl transferase gene (*bar*) for the herbicide phosphinothricin (bialaphos) resistance, and acetolactate synthase gene (*sur*) from the *Magnaporthe oryzae* for sulfonylurea (chorimuron ethyl) selection, could be used for gene disruption and genetic complementation in *B. bassiana*^19^,^20^,^21^. Therefore, only two genes could be simultaneously disrupted in the wild type *B. bassiana*, which are conducted in successive transformations. Uridine-requiring auxotrophs that lack either orotidine-5′-monophosphate pyrophosphorylase (OMPpase) (URA5) or orotidine-5′-monophosphate decarboxylase (URA3) have been widely used for various genetic screens and transformation systems in *Saccharomyces cerevisiae* and many filamentous fungi, including *Aspergillus* spp., *Trichoderma* spp. and *Cryptococcus neoformans*^22^,^23^,^24^. Recently, uridine auxotroph resulting from the disruption of *ura3* gene was obtained in *B. bassiana*. The mutants required exogenous uridine for growth and were positively selected in the medium containing 5-fluoroorotic acid (5-FOA)^25^, indicating that uridine auxotrophy can be used as a selectable marker for the transformation of *B. bassiana*.

More recently, the CRISPR (clustered regularly interspaced short palindromic repeats)-Cas9 system, as a bacterial and archaeal adaptive defense system against invading viruses and DNA^26^, has been successfully developed as an efficient genome editing tool in both prokaryotes and eukaryotes^27^,^28^,^29^. This system consists of a multidomain Cas9 endonuclease and a chimeric singleguide RNA (sgRNA) containing a 20-nt guide sequence matching with the target DNA region followed by a short Protospacer Adjacent Motif (PAM) and a downstream chimeric scaffold sequence (gRNA scaffold)^30^,^31^. The sgRNA can recruit Cas9 to create a specific DNA double strand break (DSB) in the target region, leading to deletions, insertions and substitutions through error-prone NHEJ in the absence of a homologous sequence or gene specific replacement through homologous recombination-directed repair (HDR) in the presence of a homologous DNA repair template (donor DNA)^27^,^32^. The CRISPR/Cas9 system has been adapted to several fungi, such as yeasts *Schizosaccharomyces pombe*^33^, *S. cerevisiae*^34^, filamentous ascomycete fungi *Trichoderma reesei*^35^, *Neurospora crassa*^36^, *M. oryzae*^37^ and *Aspergillus fumigatus*^38^,^39^,^40^. However, the CRISPR/Cas9 system or other genome-editing approaches have not yet been reported in entomopathogenic fungi.

The CRISPR/Cas9 systems in filamentous fungi were mainly based on low-yield transformation techniques (e.g., chemical transformation of protoplasts). The protoplast-based transformation is technically complicated and time-consuming^20^,^41^. In addition, the protoplasts are too fragile to be preserved for later uses. Many filamentous fungi such as *B. bassiana*^42^,^43^, *M. anisopliae*^44^, *Paecilomyces farinosus*^45^, *P. fiimosoroseus*^46^, *Hirsutella thompsonii*^47^ and *Verticillium lecanii*^48^ can produce blastospores through yeast-like budding in certain liquid cultures. An efficient blastospore-based transformation system has been developed for introduction of foreign genes into competent blastospores^43^. Importantly, blastospores are easily produced and can be long-term preserved at −80 °C in a state of ready-to-use.

In this study, a small gene *ura5* (699 bp of ORF) was developed as a uridine auxotrophic marker for *B. bassiana*. Coupling the use of blastospore-mediated transformation and uridine auxotrophy/*ura5* complementation, we have established a highly efficient CRISPR/Cas9-mediated gene editing system in *B. bassiana*. The use of blastospore-based transformation avoids laborious preparation of protoplasts for each transformation, and uridine auxotrophy complementation eliminates the need for using antibiotic/herbicide. Through this system, we have efficiently achieved site-specific mutations or introduced exogenous DNAs into the target sites via homologous recombination. We also demonstrated that CRISPR/Cas9 can be used for simultaneous disruption of multiple genes in a single transformation by co-transforming multiple gRNAs and relevant donor DNAs.

## Results

### Expression of codon-optimized *cas9* in *B. bassiana*

**Figure 1:**
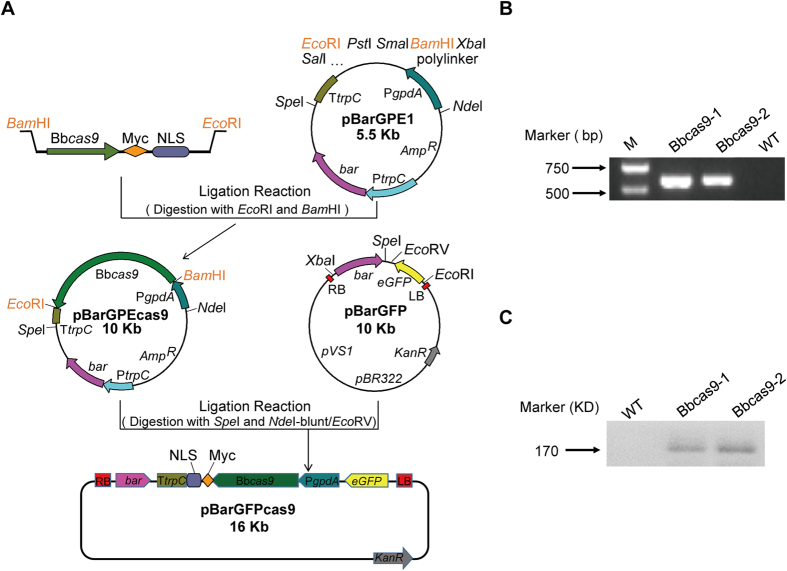
Expression of *cas9* gene in *B. bassiana* strain Bb252. (**A**) Construction of the vector expressing *cas9* under control of the *Aspergillus nidulans* constitutive promoter P*gpdA* and terminator T*trpC*. (**B**) RT-PCR analysis confirming *cas9* expression in the transformants Bbcas9-1 and Bbcas9-2. WT: wild type strain Bb252. (**C**) Detection of cas9-Myc expression in the transformants Bbcas9-1 and Bbcas9-2 by western blots.

Successful application of the CRISPR/Cas9 system in a new species requires the heterologous expression of *cas9* gene from *Streptococcus pyogenes* fused with a nuclear localization signal. The DNA sequence coding for *cas9* gene was first synthesized with *B. bassiana* preferred codon usages and cloned in frame with a C-terminal Myc tag (EQKLISEEDL) and a nuclear localization signal (PKKKRKV) (Supplementary File S1). The codon-optimized *cas9* gene (*Bbcas9*) was then placed under control of the constitutive promoter P*gpdA* and terminator T*trpC* of *Aspergillus nidulans* (Fig. 1A). The construct harboring the P*gpdA*-*Bbcas9*-T*trpC* cassette, phosphinothricin resistance gene *bar* and a marker gene coding for enhanced green fluorescent protein (eGFP) was introduced into the *B. bassiana* through *Agrobacterium*-mediated fungal transformation^14^. The expression of *Bbcas9* in the eGFP expressing transformants was first confirmed by semi-quantitative RT-PCR (Fig. 1B). The expression of Bbcas9 protein was further verified by Western blot analysis using an anti-Myc antibody. As expected, the Myc-fusion protein was detected at the predicted size, with a specific band at approximately 170 kDa in the transformants rather than in wild-type strain (Fig. 1C), suggesting that Cas9 was successfully expressed in *B. bassiana*.

Expression of the Cas9 nuclease itself without deleterious impact on *B. bassiana* growth or virulence is critical for the use of the CRISPR system in pathogenesis studies. We first tested growth rate by spotting conidia of Cas9-expressing transformants and WT strain onto SDAY plates and incubating them at 25 °C. As shown in Supplementary Fig. S1A, the colony morphology and colony diameter of the transformants were identical to that of the wild-type control, indicating that *B. bassiana* growth was unaffected by Cas9 expression. The amount of conidia produced by the Cas9-expressing transformants was also indistinguishable from that of the wild type, indicating that asexual development was also unaffected (Supplementary Fig. S1B). We next tested the virulence of the strains against host mosquitoes. No significant differences in insect survival rates were detected between Cas9-expressing transformants and wild-type strain (Supplementary Fig. S1C). In summary, constitutive expression of the Cas9 endonuclease did not influence fungal growth, conidia production, and virulence under the tested conditions. Therefore, the Cas9-expressing strain can be used as recipient for downstream sgRNA transformation and serve as universal strain for CRISPR-mediated mutagenesis.

### The *ura5* gene as a selectable marker in *B. bassiana*

Uridine-requiring auxotrophs that lack either URA5 or URA3 have been widely used for various genetic screens and transformation systems in many fungi. *B. bassiana* has single copy of the *ura5* gene based on genome sequence^49^, and the open reading frame (ORF) of *ura5* is only 699 bp long, smaller than *ura3* gene (ORF, 1,089 bp), which will facilitate the construction of donor DNAs containing an *ura5* complementation cassette. URA5 can convert 5′-fluoroorotic acid (5-FOA) into the toxic compound 5-fluorouracil (5-FU) in the form of 5-fluorouridylic acid (5-FUMP). 5-FU is a pyrimidine analogue that can be misincorporated into RNA and DNA in place of uracil or thymine, causing cell death. *ura5* mutant requires exogenous uridine for growth and could be positively selected in medium containing 5-FOA. Complementation of uridine auxotrophy by the introduction of *ura5* gene can be selected in the medium without exogenous uridine^50^. The *ura5* null mutants generated by homologous replacement via *A. tumefaciens* mediated transformation were isolated using a positive selection in M-100 medium supplemented with 20 mM uridine and 0.8 mg/ml 5-FOA. As expected, the *ΔBbura5* mutants failed to grow in the absence of uridine, but vegetative growth and full conidiation of *ΔBbura5* mutants could be recovered by adding uridine to the SDAY medium, showing no obvious difference compared to the wild-type (Supplementary Fig. S2A,B). The mutant *ΔBbura5* failed to kill insects because the insect integument does not have sufficient uridine to allow the growth of the ura5 mutant. Complementation via the introduction of *Bbura5* gene not only complemented the uridine auxotrophy of the *ΔBbura5* mutant and restored 5-FOA sensitivity, but also completely restored fungal virulence against host mosquitoes (Supplementary Fig. S2C). These data indicate that the repeatable use of this auxotrophic marker gene *ura5* is a useful genetic tool for the generation of multi-gene knockout and/or genetic complementation in *B. bassiana*.

### CRISPR/Cas9 efficiently introduces directed *URA5* mutagenesis in *B. bassiana* via nonhomologous end-joining repair

**Figure 2:**
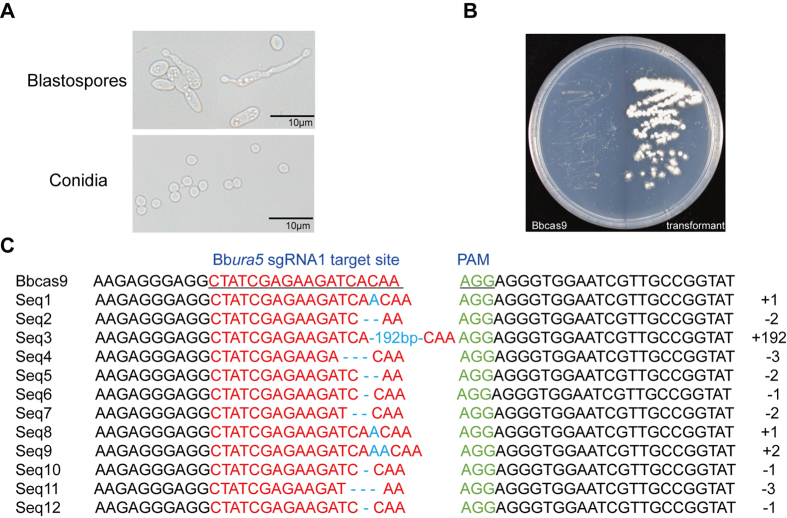
u*ra5* gene was highly efficiently disrupted in Cas9-expressing *B. bassiana* by CRISPR/Cas9 system via blastospore-based transformation. (**A**) Blastospores and conidia of the *B. bassiana*. Bar = 10 μm. (**B**) Growth of the Cas9-expressing *B. bassiana* and one representative *ura5* disrupted mutant on the M-100 plate containing 0.8 mg/ml 5-FOA and 20 mM Uridine. (**C**) Sequence alignment of the *ura5* locus of Cas9-expressing *B. bassiana* and 12 transformants (Seq1–Seq12). The PAM sequence is highlighted in green, whereas the sgRNA guiding sequence is highlighted in red. Nucleotide deletions and substitutions are depicted in blue.

An efficient CRISPR system contains both the enzyme Cas9 for cleaving DNA and sgRNA for targeting gene. The corresponding sgRNAs were transcribed *in vitro* using the MEGAscript T7 Kit (Ambion). To test the performance of the Cas9-sgRNA system in *B. bassiana*, we chose *ura5* as the first target gene because the *ura5* deficient strains have easily distinguishable phenotypes compared to WT, becoming auxotrophic for uracil or uridine, and resistant to the toxic uracil analog 5-FOA^24^,^25^,^35^. Next, the sgRNA containing a 20 bp sequence targeting the *ura5* gene in front of a PAM site (referred as *Bbura5*-sgRNA1) was designed. The competent blastospores of the Cas9-expressing *B. bassiana* were prepared as recipient cells of the exogenous sgRNA (Fig. 2A), and stored at −80 °C for later transformation. The *Bbura5*-sgRNA1 was introduced into recipient strain via blastospore-based transformation^43^. Dozens of colonies were displayed on the M-100 plates containing 0.8 mg/ml 5-FOA and 20 mM uridine. No fungal colony was observed on the control transformation plates, in which sgRNA was not used in the transformation. We randomly chose 12 transformants and transferred onto new M-100 plates containing 0.8 mg/ml 5-FOA and 20 mM uridine, and found that all the putative transformants could survive (Fig. 2B). To further validate that mutations were caused by the cleavage activity of gRNA-guided Cas9 at the endogenous loci of the target, the PCR products of *Bbura5* gene from 12 transformants were sequenced. All the selected 5-FOA-resistant transformants displayed a deletion or insertion at the expected target site, proximally located 3 bp upstream of the PAM sequence (Fig. 2C). This is consistent with the fact that CRISPR/Cas9-guided DSBs can be repaired through the NHEJ mechanism, which generates insertions and deletions in the vicinity of the cleavage site.

### CRISPR/Cas9 mediated gene replacement in *B. bassiana* via homologous recombination-directed repair

**Figure 3:**
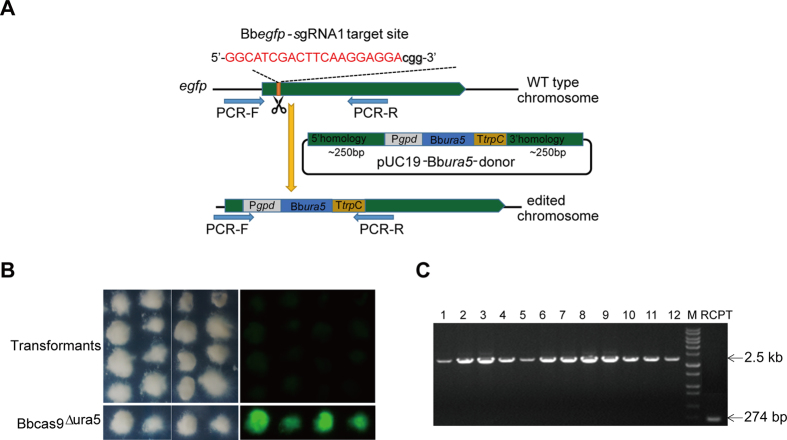
CRISPR/Cas9 mediated gene knockout through homologous recombination-directed repair (HDR) in *B. bassiana* Bb252. (**A**) Schematic showing homologous replacement of *egfp* by homologous donor DNA-mediated HDR via CRISPR/Cas9 system. (**B**) Bright-field and fluorescent images of the eGFP-expressing recipient strain Bbcas9^∆ura5^ and 16 randomly selected *egfp*-disrupted transformants via CRISPR/Cas9 gene editing system. All the transformants were non-fluorescent, indicating the *egfp* was successfully disrupted in all transformants (100% efficiency). Fungal conidia were point inoculated on SDAY plates and incubated for 2 d at 25 °C. (**C**) Replacement-specific PCR analysis. Confirmation of the predicted gene replacement was conducted with a pair of primers located on either side of homologous arms. The 274 bp and 2.5 kb bands represent the wild type and the *egfp* disruption genotype, respectively. M: DNA molecular marker; 1–12: twelve randomly selected transformants; RCPT: recipient strain Bbcas9^∆ura5^.

As the nick generated by the Cas9-gRNA complex could be sealed by the endogenous repair mechanisms NHEJ or HDR, we next attempted to test the efficiency of HDR by CRISPR/Cas9 system in *B. bassiana*. To use an endogenous *ura5* gene as a selectable marker, we used the uridine-dependent Cas9-expressing strain (named as Bbcas9^**∆**ura5^ strain) generated by *Agrobacterium*-mediated transformation as the new recipient strain. Bbcas9^**∆**ura5^ strain also expresses eGFP fluorescent marker, the successful disruption of *egfp* could be distinguished simply by fluorescent microscopy. We thus chose *egfp* gene as a target to test efficiency of using the CRISPR/Cas9 system for targeted gene replacement via homologous donor DNA-mediated HDR. To construct a homologous recombination donor DNA (dDNA) containing an *ura5* complementation cassette, the relative 250 bp homology arms of *Bbegfp* gene near the PAM site were added to both sides of the selection marker cassette (the P*gpd*-*Bbura5*-T*trpC* cassette) (Fig. 3A). After co-transformation of the *Bbegfp*-dDNA and synthesized *Bbegfp*-sgRNA1 (5′-GGCATCGACTTCAAGGAGGAcgg-3′) into the Bbcas9^**∆**ura5^ recipient, we obtained numerous transformants on the M-100 screening plates without uridine. Next we randomly chose 16 transformants and found that all transformants lost fluorescence (Fig. 3B). Further diagnostic PCR analysis confirmed that the P*gpd*-Bb*ura5*-T*trpC* selection marker cassette had integrated at the expected Cas9 cleavage site in the *egfp* locus in all tested transformants (Fig. 3C). These results demonstrated that a pair of 250 bp homology arms on either side of the target gene is sufficient to achieve efficient homologous integration stimulated by the CRISPR/Cas9 system in *B. bassiana*, compared to standard homologous integration protocol with homology arms longer than 1,000 bp.

### Simultaneous targeted mutagenesis of multiple genes in *B. bassiana* using the CRISPR/Cas9 system

**Figure 4:**
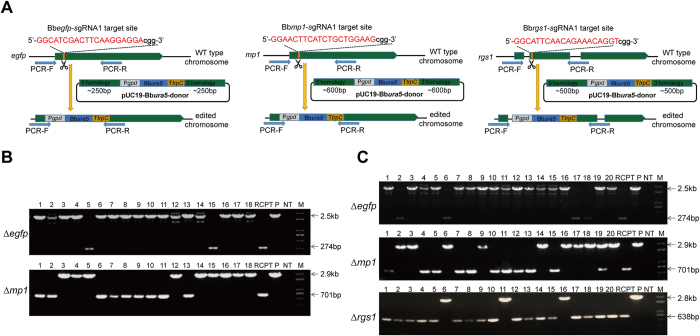
Simultaneous targeted mutagenesis of multiple genes in *B. bassiana* using the CRISPR/Cas9 system. (**A**) Schematic showing homologous replacement of *egfp, mp1* and *rgs1* by homologous donor DNA-mediated HDR via CRISPR/Cas9 system. (**B**) PCR screening of the *egfp* and *mp1* double-gene disruptants. The 274 bp and 2.5 kb bands represent the PCR products of the RCPT and the *egfp* disruptant, respectively. The 701 bp and 2.9 kb bands represent the PCR products of the RCPT and the *mp1* disruptant, respectively. (**C**) PCR screening of the *egfp, mp1* and *rgs1* triple-gene disruptants. The 274 bp and 2.5 kb bands represent the PCR products of the RCPT and the *egfp* disruptant, respectively. The 701 bp and 2.9 kb bands represent the PCR products of the RCPT and the *mp1* disruptant, respectively. The 638 bp and 2.8 kb bands represent the PCR products of the RCPT and the *rgs1* disruptant, respectively. RCPT: recipient strain Bbcas9^∆ura5^; P: plasmid as positive control; NT: no template control; M: DNA molecular marker.

Simultaneous mutagenesis of multiple genes is essential for studying functionally redundant genes and gene families. So we next assessed whether the CRISPR/Cas9 system could facilitate simultaneous multiple-locus mutagenesis in *B. bassiana*. We first attempted to simultaneously introduce two edits targeting *egfp* and a randomly selected gene *Bbmp1* (XM 008600853), coding for a putative MAP kinase, with one selection marker *ura5*. For *Bbegfp* mutagenesis, we used the same aforementioned *Bbegfp*-sgRNA1 and *Bbegfp*-dDNA. For the mutagenesis of *Bbmp1*, we designed a *Bbmp1*-sgRNA1 (5′-GGAACTTCATCTGCTGGAAGcgg-3′) and the relative *Bbmp1*-dDNA (containing P*gpd*-*Bbura5*-T*trpC* cassette and the 5′ and 3′ flanking regions of *mp1*) (Fig. 4A). Then we co-transformed the two sgRNAs and the related dDNAs into the Bbcas9^***∆***ura5^ recipient strain. One transformation yielded dozens of transformants on the uridine-free transformation plates. Diagnostic PCR analysis confirmed that 7 out of 18 (39% efficiency) randomly selected transformants were double-gene disruptants, 9 out of 18 (50%) were *egfp* single-gene disruptants, and 2 out of 18 (11%) were *mp1* single-gene disruptants (Table 1 and Fig. 4B).

Next, we tested the efficiency of simultaneous homologous replacement of triple genes. A conidiation regulatory gene *Bbrgs1* (EF116883), coding for a G protein signaling gene, was used as the third target. Mutation in *Bbrgs1* can be easily phenotypically screened for the reduced conidial yield. Similarly, the *Bbrgs1*-sgRNA1 (5′-GGCATTCAACAGAAACAGGTcgg-3′) and the relative *Bbrgs1*-dDNA (containing P*gpd*-*Bbura5*-T*trpC* cassette and the 5′ and 3′ flanking regions of *rgs1*) were generated (Fig. 4A). After co-transformation of these three sets of gRNAs and dDNAs, 20 tranformants were selected and then checked by diagnostic PCR. The results were summarized in Table 2 and Fig. 4C. There were one *∆egfp/∆mp1/∆rgs1* triple-gene disruptant (5%), four *∆egfp/∆mp1* double-gene disruptants (20%), one *∆mp1/∆rgs1* double-gene disruptant (5%), one *∆egfp/∆rgs1* double-gene disruptant (5%), ten *∆egfp* single-gene disruptants (50%) and three *∆mp1* single-gene disruptants (15%). All the *Bbrgs1* mutants showed significantly reduced conidial production on SDAY plates. Together, these results suggested that the CRISPR/Cas9 system is a reliable and powerful approach to disrupt multiple genes simultaneously in *B. bassiana*.

## Discussion

Efficient genetic manipulation approaches would greatly accelerate future progress on the systematic interrogation of gene functions in *B. bassiana* and gene engineering for mycoinsecticide improvement. Gene disruption is mainly achieved through *Agrobacterium-*mediated transformation in *B. bassiana*, but the rate of homologous recombination has been relatively low due to the predominant employment of the NHEJ repair pathway, which makes reverse genetics challenging. Although homologous recombination rate could presumably be increased with larger flanking sequences, the cloning efficiency becomes the limiting factor. Therefore, standard gene deletion/disruption protocols by homologous integration typically use flanking regions of longer than 1 kb on either side of the target gene.

Our study demonstrated that the CRISPR/Cas9 system showed very high frequency of homologous recombination when a pair of 250 bp flanking homology arms were used. The introduction of foreign DNA into *B. bassiana* usually relies on fungal protoplasts via PEG-mediated DNA transformation, electroporation^20^ and gene gun bombardment^41^ or conidia via *Agrobacterium*-mediated transformation^14^,^21^. The first three approaches usually suffer from low transformation efficiency and laborious preparation of protoplasts, which are too fragile to be preserved in laboratory for future uses^20^,^41^. The *Agrobacterium*-mediated transformation is time-consuming and less useful for gene replacement through homology-directed repair^18^. We have developed the CRISPR/Cas9 system in *B. bassiana* based on blastospore transformation instead of protoplast transformation. The competent blastospores are easily prepared and can be frozen for long-term storage^43^, which will be applicable to other blastospore-producing fungi. So far, gene disruption or replacement has relied on the use of antibiotic or herbicide resistance markers. In this study, we developed a markerless transformation system for *B. bassiana* by exploiting uridine auxotrophy, resulting from the disruption of the *ura5* gene. The size of *ura5* gene (699 bp) is smaller than *ura3* gene (1,098 bp), which facilitates construction of donor DNAs for the complementation of uridine auxotrophy. Consequently, the target gene was replaced by *ura5* restoring the ability of the transformants to grow in the absence of exogenous uridine and the sensitivity to 5-FOA. Since the *ura5* mutant cannot grow without uridine, our gene editing system based on uridine auxotrophy/*ura5* complementation has thus low false-positive background. Moreover, the lack of the need for using antibiotic/herbicide is also cost-effective. More importantly, our system has allowed simultaneous editing of multiple unrelated genes in a single transformation. Although the frequency for double-replacement mutant is 32% and for triple-replacement mutant is only 5% in the tested genes, we have reasons to believe that a higher frequency of simultaneous targeting multiple genes can be achieved by optimizing the proportion of the different gRNAs and dDNAs of multiple genes^35^ or even using different donors containing various selection markers. We note that introducing the *ura5* cassette into multiple target gene loci cause over-expression of *ura5* in the mutant strains. Although we did not observed obvious change in vegetative growth and conidia production between the ∆egfp/∆mp1 double mutants and WT, effect of *ura5* overexpression on virulence of *B. bassiana* should be tested in a future study. In addition, an inducible promoter or various selectable marker genes will also be tested.

This study demonstrates precise editing of *B. bassiana* genome through the repair of Cas9-induced DSBs in an NHEJ-mediated error-prone DNA repair and HDR-mediated error-free DNA replacement. Although several studies in mammalian cells and other systems have reported that Cas9/gRNA complexes have the ability to bind and cut DNA sequences with imperfect homology, thereby giving rise to off-target mutations^51^, several mismatches scattered in the binding region, especially the sequence closest to the PAM (also called the ‘seed sequence’), appears to eliminate mutagenesis^52^,^53^. Hence, off-target effects may be problematic in large-genome organisms. Indeed, so far off-target mutations of CRISPR/Cas9 have not been detected in *Drosophila*^54^,^55^. Whole genome sequencing of *S. cerevisiae* strains that were mutated via CRISPR/Cas9 system also indicated that off-target effects are insignificant in fungi^56^,^57^. To minimize the off-target effects of Cas9 in this study, we tried to avoid selection of target sites, especially the 12 nucleotides proximal to the protospacer, which have closely homologous sequences elsewhere in the *B. bassiana* genome.

In summary, by combining the use of blastospore-mediated transformation and uridine auxotrophy/*ura5* complementation, we have demonstrated that CRISPR/Cas9 can be a powerful tool for high-efficiency targeted gene knock-out and /or knock-in in *B. bassiana*, and holds significant potential for advancing understanding of its pathogenesis. More importantly, our system will allow us to address the issue of functional redundancy where generating disruptions of three genes is limited by availability of only two resistance markers. The development and successful application of the CRISPR/Cas9 system for genome editing in *B. bassiana* may have broad applications to other entomopathogenic fungi.

## Materials and Methods

### Strains and plasmids

*E. coli* DH5α was used as a host for plasmid cloning and maintenance. The *B. bassiana* strain 252 (Bb252) was used as the host for Cas9 expression. *A. tumefaciens* AGL-1 was used to transform the *cas9* gene to Bb252. Fungal strains were maintained on Sabouraud Dextrose Agar plus Yeast extract (SDAY, BD Difco) at 25 °C. pMD-18T vector (Takara, Dalian, China) was used to maintain gRNA template and PUC19 vector (Takara, Dalian, China) was used to maintain dDNAs in DH5a. pBarGPE and pBarGFP were used to construct the transformation vectors.

### Expression of cas9 in *B. bassiana*

Primers used for plasmid construction and templates synthesis were listed in Supplementary Table S1. Primers used for diagnostic PCR and sequencing analysis were listed in Supplementary Table S2. To generate the Cas9-expressing plasmid pBarGFPcas9, where Cas9-Myc-NLS is placed under the control of the constitutive promoter P*gpdA*, we synthesized a *B. bassiana* codon-optimized *cas9* gene from *Streptococcus pyogenes*. The synthesized gene was designed in frame with a C-terminal Myc tag (EQKLISEEDL) and a nuclear localization signal peptide (PKKKRKV). The 4,209 bp DNA fragment containing Cas9-Myc-NLS was cloned into the *Bam*HI and *Eco*RI sites of the plasmid pBarGPE1^58^, which contains a *gpdA* promoter (P*gpdA*) and a *trpC* terminator (T*trpC*) flanking the *Bam*HI and *Eco*RI sites. The P*gpdA*-*Bbcas9*-T*trpC* cassette was excised from pBarGPEcas9 as a 6,423 bp of *Spe*I/ *Nde*I-blunt fragment and ligated into the corresponding *Spe*I/*Eco*RV sites of pBarGFP^59^ to generate pBarGFPcas9. pBarGFPcas9 was transformed into wild type Bb252 using *Agrobacterium*-mediated fungal transformation. Transformants were selected on M-100 plate^60^ containing glufosinate-ammonium (200 μg/ml) and cefotaxime (400 μg/ml).

For preparation of RNA, the fungal spores were cultured on cellophane in SDAY medium for 3 days. Then the samples were collected and homogenized in liquid nitrogen, and total RNA from *B. bassiana* was extracted using RNAiso Plus (Takara). cDNA of each sample was generated using PrimeScript^TM^ RT reagent Kit with gDNA Eraser (Takara). Complementary DNA samples were used as template for RT-PCR to verify *Bbcas9* gene expression. The primers are listed in Supplementary Table S2.

For preparation of fungal proteins, the transformants and wild type strains were grown on SDAY for one week and then the conidia were cultured in Sabouraud dextrose broth (SDB) (10^6^ conidia/ml) for 36 h at 25 °C. The mycelium collected by filtration was ground in liquid nitrogen and suspended in RIPA buffer (Beyotime, P0013B). Then the protein was examined by SDS-PAGE and western blots using an anti-Myc antibody (abmart, M20002).

### Disruption of *ura5* via *A. tumefaciens-*mediated homologous recombination

For targeted disruption of *ura5*, the 5′and 3′flanking regions of the *ura5* ORF were amplified by PCR from Bb252 genomic DNA, and then subcloned into the *Xba*I and *Spe*I sites of the binary vector pBarGFP. The gene disruption construct (pBarGFPura5) was then transformed into *A. tumefaciens* AGL-1 for targeted gene disruption via homologous recombination. *ΔBbura5* mutants were positively selected on M-100 plates supplemented with 0.8 mg/ml 5-FOA and 20 mM uridine.

### Insect bioassays

Fungal virulence bioassays were conducted against 3–5 day-old female *Anopheles stephensi* mosquitoes. The mosquitoes were anesthetized by carbon dioxide (CO_2_) and topically inoculated by spraying 5 × 10^7^ conidia/ml suspension with 1 ml glass atomizer. The excess liquid on the mosquito bodies was removed by placing them on dry paper towels. Controls were treated with sterile 0.01% Triton X-100 solution. Experiment and control mosquitoes were placed in cups and maintained on 10% sucrose solution at 27 °C and 80 ± 5% relative humidity. Each treatment was replicated three times with 50 female mosquitoes per replication, and the bioassays were repeated three times. Insect mortality was recorded every 12 h after treatment.

### Design and synthesis of sgRNAs by *in vitro* transcription

gRNAs were designed by manually searching gene regions for the presence of Protospacer-Adjacent Motifs (PAMs) with the sequence NGG, where N was any nucleotide. We set out the conditions under which gRNA sequence was 20 bp in length, excluding the PAM, and contained one or two 5′ terminal guanines to facilitate transcription by T7 RNA polymerase. To minimize the potential for off-target mutagenesis, we avoided gRNA sequences with closely related binding sites to reduce the possibility of cutting. The gRNA cassette containing a 17 bp T7 promoter sequence, a 20 bp gRNA sequence and an 80 bp gRNA scaffold was synthesized. This cassette was then ligated into pMD-18T and maintained in DH5α for the gRNAs transcription. Following the manufacturer′s instructions, the amplified product from pMD-18T purified by Gel Extraction Kit (Omega) was transcribed into RNA *in vitro* using the MEGAscript T7 Kit (Ambion, Life Technologies). Then, the transcribed gRNA was purified by phenol/chloroform extraction solution followed by isopropanol precipitation. The purified RNA was dissolved in nuclease-free water.

### Construction of donor DNA for target gene ‘knock-in’ replacement

For construction of the plasmid pUC19-Bb*ura5*-Donor, the *gpd* promoter (P*gpd*) fragment and the *trpC* terminator (T*trpC*) fragment were PCR-amplified from *Cordyceps militaris* genomic DNA with primer pairs Pgpd-F/Pgpd-R and Ttrpc-F/Ttrpc-R, respectively. The *ura5* gene was amplified from the genomic DNA of Bb252 using primers Bbura5-F and Bbura5-R. These three fragments were sequentially assembled with the ClonExpress^TM^ MultiS one step cloning kit (Vazyme, C113-01), and cloned into the *Sma*I site of the plasmid pUC19 to generate the plasmid pUC19-Bb*ura5*-Donor. To construct dDNAs of different genes, the 5′ and 3′ flanking sequences of target genes were amplified and cloned into the *Sma*I site of the plasmid pUC19-Bb*ura5*-Donor vector. The generated vectors were propagated in DH5α and purified using the Plasmid Midi Kit (Omega).

### Blastospore-based fungal transformation

Preparation of blastospores and competent blastospore-based transformation were performed according to a previously described method^43^ with some modifications. Briefly, fungal conidia were inoculated in SDB broth and shaken at 120 rpm at 25 °C for 2 days. Each 5 ml aliquot of the culture was transferred into 50 ml GM medium (w/v, 4% glucose, 0.4% NH_4_NO_3_, 0.3% KH_2_PO_4_, and 0.3% MgSO_4_) and cultured for 24–30 h. The formed blastospores were collected by filtering through four-layer lens papers and concentrated by 3,000 rpm centrifugation at 4 °C for 5 min. The harvested blastospores were washed twice with sterile ddH_2_O by centrifugation at 3,000 rpm for 5 min at 4 °C, and then suspended in 0.1 M LiAc (1:50). This blastospore suspension was transferred into 1.5 ml Eppendorf tubes, precipitated by centrifugation at 7,000 rpm for 5 min at 4 °C, and then re-suspended in 0.1 M LiAc (1:100). Samples were incubated at 4 °C for 10 min, then supplemented in 12% glycerol at a final concentration of 3 × 10^8^ cells/ml and immediately frozen (liquid nitrogen) in 100 μl aliquots and stored at −80 °C.

For transformation, all operations were conducted at 4 °C using ice-cold solutions. The frozen competent cells were thawed on ice, and harvested by centrifugation at 7,000 rpm for 5 min. The following agents were added in the following order: 240 μl 50% PEG 4000, 36 μl of 1 M LiAc, 50 μl of 2 g/L denatured genomic salmon sperm DNA, 50 μg sgRNA fragments and 20 μg donor DNA-containing plasmid, and 35 μl of 1 M dithiothreitol. Then the samples were thoroughly mixed for 1 min after addition of the last reagent. The samples were incubated in ice for 30 min, then subjected to heat shock for 20 min at 42 °C and immediately incubated in ice for 5 min. The cells were harvested by centrifugation at 7,000 rpm for 5 min at 4 °C and resuspended in 0.2 ml of sterile distilled H_2_O at room temperature. The targeted transformants were selected on the M-100 medium without exogenous uridine.

### Diagnostic PCR and DNA sequencing analysis

The putative knock-in mutants generated by CRISPR/Cas9 system were verified by diagnostic PCR or sequencing analysis. The fungal genomic DNAs were extracted according to the standard protocol (phenol/chloroform extraction and isopropanol precipitation). Primers for indel analysis were designed to cover upstream and downstream of the expected gRNA cleavage sites.

## Additional Information

**How to cite this article:** Chen, J. *et al*. CRISPR/Cas9-mediated efficient genome editing via blastospore-based transformation in entomopathogenic fungus *Beauveria bassiana. Sci. Rep.* **7**, 45763; doi: 10.1038/srep45763 (2017).

**Publisher's note:** Springer Nature remains neutral with regard to jurisdictional claims in published maps and institutional affiliations.

## Supplementary Material

Supplemental Information

## Figures and Tables

**Table 1:** Transformants from double-gene disruption of *egfp* and *mp1* in *B. bassiana*.

Mutants	No. of transformants	HR frequency
∆egfp/∆mp1	7	39%
∆egfp	9	50%
∆mp1	2	11%

**Table 2:** Transformants from triple-gene disruption of *egfp, mp1* and *rgs1* in *B. bassiana*.

Mutants	No. of transformants	HR frequency
∆egfp/∆mp1/∆rgs1	1	5%
∆egfp/∆mp1	4	20%
∆mp1/∆rgs1	1	5%
∆egfp/∆rgs1	1	5%
∆egfp	10	50%
∆mp1	3	15%
