# Supplementary material for: CRISPR/Cas9-mediated efficient genome editing via blastospore-based transformation in entomopathogenic fungus Beauveria bassiana
Source: Sci Rep. 2017 Apr 3;7:45763. doi: 10.1038/srep45763 (PMC5377935; doi:10.1038/srep45763)
Supplement: Supplemental Information [file srep45763-s1.pdf]

Supplementary Information

CRISPR/Cas9-mediated efficient genome editing via blastospore-based transformation in entomopathogenic fungus *Beauveria bassiana*

Jingjing Chen[1,2], Yiling Lai[1], Lili Wang[1,2], Suzhen Zhai[1], Gen Zou[3], Zhihua Zhou[3], Chunlai Cui[1,2], Sibao Wang[1]*

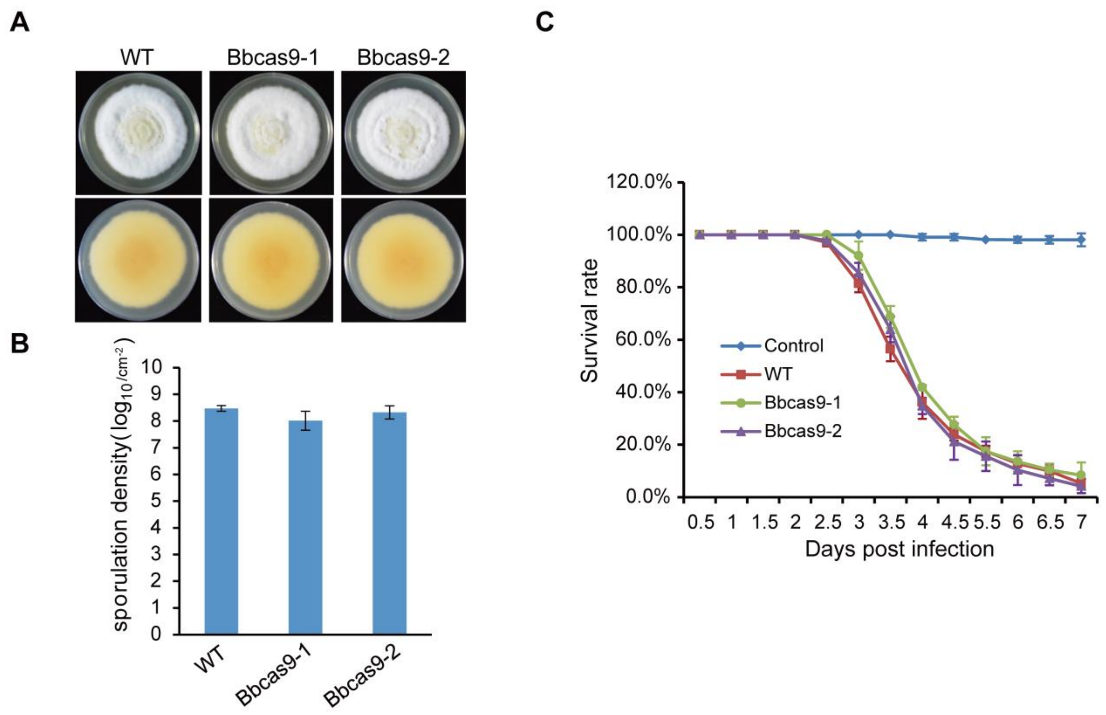

Supplementary Figure S1. Expression of the *cas9* does not impact *B. bassiana* growth and virulence. Growth (A), conidia production (B) and virulence (C) analysis of the wild-type Bb252 and two randomly selected Cas9-expressing strains. Conidia of the indicated strains were point-inoculated on SDAY for 12 days. The adult *Anopheles stephensi* mosquitoes were topically inoculated with conidia suspension. There were no significant differences ($P = 0.43$, t-test) in conidia production and fungal virulence between WT and transformants. Error bars represent the standard deviation among three replicates. Control: treated with sterile 0.01% Triton X-100 solution without conidia.

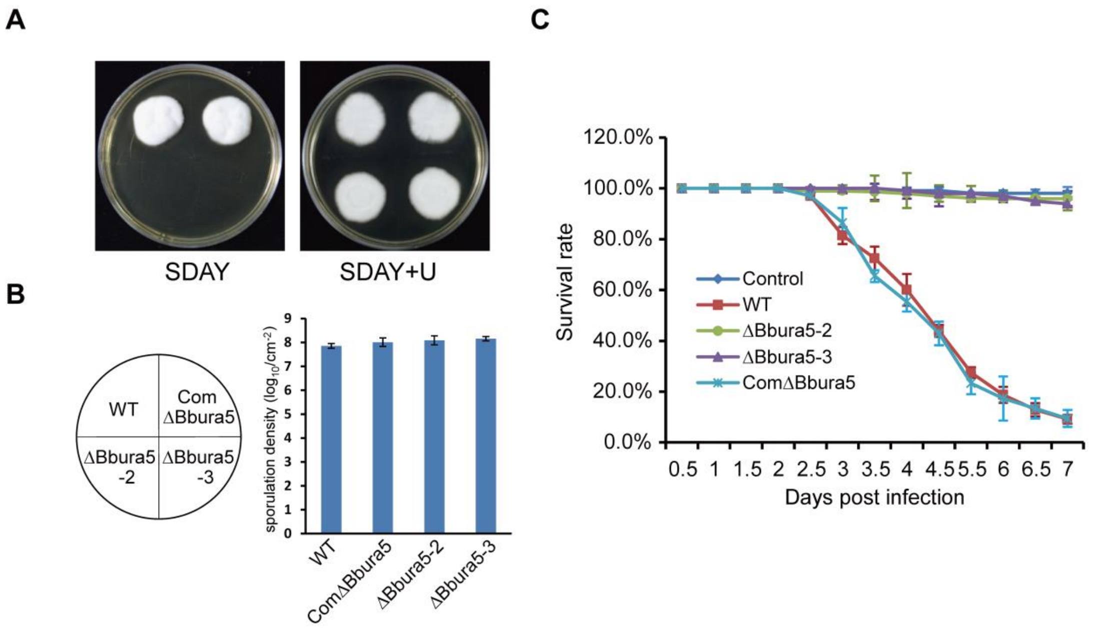

Supplementary Figure S2. Growth, conidia production and virulence of *B. bassiana* wild-type (WT), mutant ΔBbura5 and complemented strain ComΔBbura5. (A) Colony morphology after 7 days growth on SDAY and SDAY supplemented with uridine (U), respectively. (B) Conidial yield after 7 days of growth on SDAY supplemented with uridine (number of conidia per square centimeters). (C) Survival rate of the adult *Anopheles stephensi* mosquitoes after topical infection. Control: treated with sterile 0.01% Triton X-100 solution without conidia.

Supplementary Table S1. Primers used for plasmid construction and donor templates synthesis in this study.

**Primer name**	**Sequence(5′ to 3′)**	**Application**
Pgpd-F	TCGAGCTCGGTACCCGGGTGAGGCTGGTGAATATGACTAG	Cloning of *gpd* promoter
Pgpd-R	AGCGAGTTGCGACATTGTTCTTGATTAGAAAAGTGAGG	Cloning of *gpd* promoter
Ttrpc-F	AAGGCCAGCGACTGAGATGAGGACTCCTCAGCTA	Cloning of *trpc* terminator
Ttrpc-R	CTCTAGAGGATCCCCGGGAAGTGATGAGGGGACAAGTCT	Cloning of *trpc* terminator
Bbura5-F	ATGTCGCAACTCGCTCAGTAC	Cloning of Bb*ura5*
Bbura5-R	TCAGTCGCTGGCCTTATACTT	Cloning of Bb*ura5*
gRNA-scaffold-R	AAAAGCACCGACTCGGTGCCACTTTTTCAAGTTGATAACGGACTAGCCTTATTTTAACTTGCTATTTCTAGCTCTAAAAC	Synthesizing the gRNA Cloning fragment
sgRNA-egfp-F	TAATACGACTCACTATAGGCATCGACTTCAAGGAGGAGTTTTAGAGCTAGAAATAGC	Synthesizing the gRNA Cloning fragment of *egfp*
sgRNA-mp1-F	TAATACGACTCACTATAGGCCGTCAGCAAGATCCCCAGTTTTAGAGCTAGAAATAGC	Synthesizing the gRNA Cloning fragment of *mp1*
sgRNA-rgs1-F	TAATACGACTCACTATAGGCATTCAACAGAAACAGGTGTTTTAGAGCTAGAAATAGC	Synthesizing the gRNA Cloning fragment of *rgs1*
sgRNA-tail-PCR-F	TCGGTGATGACGGTGAAAA	Amplifying the template of sgRNA
sgRNA-tail-PCR-R	CGTGGCTCAGCCACGAAAA	Amplifying the template of sgRNA
egfp-donor-LF	AATTCGAGCTCGGTACCCGGGCTCGTGACCACCCTGACCTA	Cloning the 5′ flanking region of *egfp*
egfp-donor-LR	CTGGCTTAACTATGCGGCCTCGATGCGGTTCACCAG	Cloning the 5′ flanking region of *egfp*
egfp-donor-RF	ATTCCGTCACCAGCCCTGAAGCTGGAGTACAACTACAACAGC	Cloning the 3′ flanking region of *egfp*
egfp-donor-RR	CGACTCTAGAGGATCCCCGGGCGAACTCCAGCAGGACCAT	Cloning the 3′ flanking region of *egfp*
mp1-donor-LF	AATTCGAGCTCGGTACCCGGGTGCCAACAAAAGTAACCG	Cloning the 5′ flanking region of *mp1*
mp1-donor-LR	CTGGCTTAACTATGCGGCGCTTGTCCTTGAGGTCGT	Cloning the 5′ flanking region of *mp1*

mp1-donor-RF	ATTCCGTCACCAGCCCTGCGTCCACTAGCTCCCAGAC	Cloning the 3′ flanking region of *mp1*
mp1-donor-RR	CGACTCTAGAGGATCCCCGGGCCCTGACTCCGCACTACAT	Cloning the 3′ flanking region of *mp1*
rgs1-donor-LF	AATTCGAGCTCGGTACCCGGGTGGCGTCCTTTGTTTGCT	Cloning the 5′ flanking region of *rgs1*
rgs1-donor-LR	CTGGCTTAACTATGCGGCCATGGTGTAGACCTGCTGGTAC	Cloning the 5′ flanking region of *rgs1*
rgs1-donor-RF	ATTCCGTCACCAGCCCTGCTACCACCAGCAACCTCCAA	Cloning the 3′ flanking region of *rgs1*
rgs1-donor-RR	CGACTCTAGAGGATCCCCGGGGGACGAACAAGCTGGCAAT	Cloning the 3′ flanking region of *rgs1*
Bbura5-FF	CAGTACACGAGGACTTCTAGAATGCTGGCGAACCTGGTCA	Cloning the 5′ flanking region of *ura5*
Bbura5-FR	TCATCTTCTGTCGAGTCTAGAGCGATCGGTCAAGTTCTTTTCT	Cloning the 5′ flanking region of *ura5*
Bbura5-RF	GTGTCTGTATTTCCGGATATCGATGTAGGCTCAATACCGGGG	Cloning the 3′ flanking region of *ura5*
Bbura5-RR	ACCGACGGAATTGAGGATATCCGTTGGCGGTGTTAGGATGT	Cloning the 3′ flanking region of *ura5*
sgRNA-ura5-F	TAATACGACTCACTATAGGCTATCGAGAAGATCACAAGTTTTAGAGCTAGAAATAGC	Synthesizing the gRNA cloning fragment of ura5

Table S2. Primers for diagnostic PCR screening and sequencing analysis in this study.

Name	Sequence(5′ to 3′)	Application
pBarGPE-cas9-F	GTGTATGAAACCGGAAAGGC	Verifying the *cas9* gene
pBarGPE-cas9-R	GATCTCCACAGAATCGAAGCA	Verifying the *cas9* gene
Bbcas9-RT-PCR-F	AAGGGCCGTGATTTCGC	Verifying the *cas9* gene
Bbcas9-RT-PCR-F	CGTCCAGGTAATGCTTGTGC	Verifying the *cas9* gene
M13-47-*F*	CGCCAGGGTTTTCCCAGTCACGAC	Sequencing the template of sgRNA synthesized in vitro
RV-M-R	GAGCGGATAACAATTTCACACAGG	Sequencing the template of sgRNA synthesized in vitro
Bbura5-seq-F	ATGTCGCAACTCGCTCAGTAC	Sequencing and verifying Bb*ura5*
Bbura5-seq-R	TCAGTCGCTGGCCTTATACTT	Sequencing and verifying Bb*ura5*
Donor-seq-1F	TACACTCCGCTATCGCTACG	Sequencing and verifying the Pgpd-Bb*ura5*-Ttrpc cassette
Donor-seq-2F	TCTGTAGGGCGTCCAAATATC	Sequencing and verifying the Pgpd-Bb*ura5*-Ttrpc cassette
Donor-seq-3F	AAGGCTTTTGCCCTCACC	Sequencing and verifying the Pgpd-Bb*ura5*-Ttrpc cassette
Donor-seq-4F	ATTTCCGAGGAGGACATCAA	Sequencing and verifying the Pgpd-Bb*ura5*-Ttrpc cassette
Donor-seq-1R	CGTTGATCTGCTTGATCTCGTC	Sequencing and verifying the Pgpd-Bb*ura5*-Ttrpc cassette
Donor-seq-2R	TGCGGTCAAACGAGTAGGAA	Sequencing and verifying the Pgpd-Bb*ura5*-Ttrpc cassette
Donor-seq-3R	TCTTTTACCAGATCGGAAGCA	Sequencing and verifying the Pgpd-Bb*ura5*-Ttrpc cassette
Bbegfp-seq-F	TGCTTCAGCCGCTACCC	Verifying the *egfp* gene
Bbegfp-seq-R	TGCCGTTCTTCTGCTTGTC	Verifying the *egfp* gene
Bbmp1-seq-F	CGCATCCGTCCTTACCC	Verifying the *mp1* gene
Bbmp1-seq-R	CTGCTCCCATAGCCCATAC	Verifying the *mp1* gene
Bbrgs1-seq-F	TTGGTTACCTCTTCGCCTATC	Verifying the *rgs1* gene

Bbrgs1-seq-R	GACCGTCCTGGTAATCGTG	Verifying the *rgs1* gene
Bbura5-midR	CAGCGAGGCGAGCAGTAT	Verifying the *ura5* gene
BarF1	CACGGGAACTGGCATGA	Verifying the *ura5* gene
Bbura5-RT-PCR_F	CGAGGCTCAGAAGAATGGC	Verifying the *ura5* gene
Bbura5-RT-PCR_R	GAGAAAATGGGAATGCCGTA	Verifying the *ura5* gene

> *B. bassiana* codon-optimized *cas9*-Myc-NLS
ATGGACAAGAAGTACAGCATCGGCCTGGATATTGGCACTAACAGCGTGGG
CTGGGCGGTCATCACCGACGAGTACAAGGTGCCGTCTAAGAAGTTCAAGG
TCCTGGGCAACACCGACCGCCACAGCATCAAGAAGAACCTGATTGGCGCG
CTGCTGTTCGACTCCGGCGAGACTGCTGAGGCTACCCGTCTGAAGCGTAC
GGCTCGCCGTCGCTACACTCGTCGCAAGAACCGCATCTGCTACCTCCAGG
AGATTTTCAGCAACGAGATGGCCAAGGTGGACGATTCCTTCTTCCACCGC
CTGGAGGAGTCGTTCCTGGTCGAGGAGGACAAGAAGCACGAGCGCCATC
CCATCTTCGGCAACATTGTGGACGAGGTCGCCTACCACGAGAAGTACCCA
ACTATCTACCATCTGCGCAAGAAGCTGGTGGACTCCACCGATAAGGCTGA
CCTGCGTCTGATCTACCTGGCCCTGGCTCACATGATTAAGTTCCGCGGCCA
TTTCCTGATCGAGGGCGATCTGAACCCTGACAACTCGGATGTGGACAAGC
TGTTCATCCAGCTGGTCCAGACCTACAACCAGCTGTTCGAGGAGAACCCG
ATTAACGCTTCCGGCGTGGACGCTAAGGCTATCCTGTCGGCTCGTCTGAGC
AAGTCCCGTCGCCTGGAGAACCTGATCGCCCAGCTGCCTGGCGAGAAGAA
GAACGGCCTGTTCGGCAACCTGATTGCTCTGTCTCTGGGCCTGACGCCGA
ACTTCAAGAGCAACTTCGATCTGGCCGAGGACGCTAAGCTCCAGCTGTCC
AAGGACACCTACGACGATGACCTGGATAACCTGCTGGCGCAGATCGGCGA
TCAGTACGCCGACCTGTTCCTGGCCGCTAAGAACCTGTCCGACGCTATCCT
GCTGTCGGATATTCTGCGCGTGAACACGGAGATTACTAAGGCGCCCCTGT
CGGCCTCTATGATCAAGCGTTACGACGAGCACCATCAGGATCTGACCCTG
CTGAAGGCGCTGGTGCGTCAGCAGCTGCCAGAGAAGTACAAGGAGATTTT
CTTCGATCAGTCGAAGAACGGCTACGCTGGCTACATTGACGGCGGCGCGT
CTCAGGAGGAGTTCTACAAGTTCATCAAGCCCATTCTGGAGAAGATGGAC
GGCACTGAGGAGCTGCTGGTGAAGCTGAACCGCGAGGACCTGCTGCGCA
AGCAGCGTACCTTCGATAACGGCAGCATCCCCCACCAGATTCATCTGGGC
GAGCTGCACGCCATCCTGCGTCGCCAGGAGGACTTCTACCCATTCCTGAA
GGATAACCGCGAGAAGATCGAGAAGATTCTGACCTTCCGTATCCCTTACT
ACGTCGGCCCGCTGGCCCGCGGCAACAGCCGCTTCGCTTGGATGACCCGC
AAGTCGGAGGAGACTATCACCCCCTGGAACTTCGAGGAGGTGGTGGACA
AGGGCGCTTCGGCGCAGTCTTTCATTGAGCGCATGACCAACTTCGACAAG
AACCTGCCCAACGAGAAGGTGCTGCCAAAGCACAGCCTGCTGTACGAGTA
TTTCACCGTCTACAACGAGCTGACGAAGGTGAAGTACGTCACTGAGGGTA
TGCGCAAGCCGGCTTTCCTGTCCGGCGAGCAGAAGAAGGCGATCGTGGAC
CTGCTGTTCAAGACGAACCGCAAGGTCACTGTCAAGCAGCTGAAGGAGGA
CTACTTCAAGAAGATTGAGTGCTTCGATTCTGTGGAGATCAGCGGCGTCG
AGGACCGTTTCAACGCCTCCCTGGGCACCTACCACGACCTGCTGAAGATC
ATTAAGGATAAGGACTTCCTGGACAACGAGGAGAACGAGGACATCCTGG
AGGACATTGTGCTGACGCTGACTCTGTTCGAGGACCGCGAGATGATCGAG
GAGCGTCTGAAGACGTACGCGCATCTGTTCGATGACAAGGTCATGAAGCA
GCTGAAGCGTCGCCGTTACACTGGCTGGGGCCGCCTGAGCCGTAAGCTGA
TCAACGGCATTCGCGACAAGCAGTCTGGCAAGACGATCCTGGACTTCCTG
AAGAGCGATGGCTTCGCTAACCGCAACTTCATGCAGCTGATTCACGATGA
CTCGCTGACCTTCAAGGAGGACATCCAGAAGGCTCAGGTGTCTGGCCAGG

GCGACAGCCTGCACGAGCATATCGCGAACCTGGCCGGCAGCCCTGCGATC
AAGAAGGGCATCCTCCAGACCGTGAAGGTGGTGGACGAGCTGGTGAAGG
TCATGGGCCGCCACAAGCCGGAGAACATCGTCATTGAGATGGCCCGTGAG
AACCAGACCACGCAGAAGGGCCAGAAGAACTCGCGCGAGCGTATGAAGC
GCATCGAGGAGGGCATTAAGGAGCTGGGCAGCCAGATCCTGAAGGAGCA
CCCGGTGGAGAACACCCAGCTCCAGAACGAGAAGCTGTACCTGTACTACC
TCCAGAACGGCCGCGATATGTACGTGGACCAGGAGCTGGATATTAACCGT
CTGAGCGATTACGACGTGGATCATATCGTCCCTCAGTCTTTCCTGAAGGAT
GACAGCATTGACAACAAGGTGCTGACCCGCTCCGACAAGAACCGTGGCA
AGTCCGATAACGTCCCCTCGGAGGAGGTGGTCAAGAAGATGAAGAACTA
CTGGCGCCAGCTGCTGAACGCCAAGCTGATCACCCAGCGCAAGTTCGACA
ACCTGACGAAGGCTGAGCGTGGCGGCCTGTCGGAGCTGGACAAGGCTGG
CTTCATCAAGCGCCAGCTGGTGGAGACGCGTCAGATCACTAAGCACGTCG
CTCAGATTCTGGACAGCCGTATGAACACGAAGTACGATGAGAACGACAA
GCTGATCCGCGAGGTGAAGGTCATTACTCTGAAGTCCAAGCTGGTGTCGG
ACTTCCGCAAGGATTTCCAGTTCTACAAGGTCCGTGAGATCAACAACTAC
CACCATGCTCACGACGCTTACCTGAACGCTGTGGTCGGCACGGCTCTGATT
AAGAAGTACCCCAAGCTGGAGAGCGAGTTCGTGTACGGCGACTACAAGG
TGTACGATGTCCGCAAGATGATCGCCAAGTCTGAGCAGGAGATTGGCAAG
GCCACCGCTAAGTATTTCTTCTACTCCAACATCATGAACTTCTTCAAGACC
GAGATCACGCTGGCGAACGGCGAGATCCGCAAGCGTCCGCTGATTGAGAC
CAACGGCGAGACGGGCGAGATCGTGTGGGACAAGGGCCGTGATTTCGCC
ACCGTGCGTAAGGTCCTGTCCATGCCCCAGGTGAACATTGTCAAGAAGAC
TGAGGTGCAGACCGGCGGCTTCAGCAAGGAGTCCATTCTGCCAAAGCGCA
ACTCGGACAAGCTGATCGCCCGTAAGAAGGACTGGGACCCGAAGAAGTA
CGGCGGCTTCGACTCCCCAACGGTGGCGTACTCGGTCCTGGTGGTCGCCA
AGGTGGAGAAGGGCAAGTCTAAGAAGCTGAAGAGCGTCAAGGAGCTGCT
GGGCATCACCATTATGGAGCGCAGCTCCTTCGAGAAGAACCCTATCGACT
TCCTGGAGGCCAAGGGCTACAAGGAGGTGAAGAAGGACCTGATCATTAA
GCTGCCGAAGTACAGCCTGTTCGAGCTGGAGAACGGCCGTAAGCGTATGC
TGGCGTCCGCCGGCGAGTTGCAGAAGGGCAACGAGCTGGCTCTGCCTTCG
AAGTACGTGAACTTCCTGTACCTGGCGTCTCACTACGAGAAGCTGAAGGG
CAGCCCGGAGGACAACGAGCAGAAGCAGCTGTTCGTCGAGCAGCACAAG
CATTACCTGGACGAGATCATTGAGCAGATTTCGGAGTTCTCTAAGCGCGT
GATCCTGGCTGACGCGAACCTGGATAAGGTCCTGTCCGCGTACAACAAGC
ACCGCGACAAGCCCATCCGTGAGCAGGCCGAGAACATCATTCATCTGTTC
ACCCTGACGAACCTGGGCGCGCCAGCGGCCTTCAAGTATTTCGACACTAC
CATCGATCGCAAGCGTTACACTAGCACCAAGGAGGTGCTGGACGCCACCC
TGATCCACCAGTCCATTACCGGCCTGTACGAGACGCGTATCGACCTGTCG
CAGCTGGGCGGCGATTCTCGTGCTGATGGCTCGCCCGGCGAGCAGAAGCT
CATTTCGGAGGAGGACCTGGGCAGCCCTGGCCCAAAGAAGAAGCGCAAG
GTCTAA

Supplementary File S1. Sequence of the *B. bassiana* codon-optimized

Cas9-Myc-NLS. The sequence of the Cas9-Myc-NLS was optimized with *B. bassiana* preferred coding usage (http://www.kazusa.or.jp/codon/cgi-bin/showcodon.cgi?species=176275). The Myc tag sequences are highlighted in green and the nucleoplasmin NLS sequences are highlighted in blue.
